# The Changing Epidemiology of Meningococcal Disease in Quebec, Canada, 1991–2011: Potential Implications of Emergence of New Strains

**DOI:** 10.1371/journal.pone.0050659

**Published:** 2012-11-29

**Authors:** Rodica Gilca, Geneviève Deceuninck, Brigitte Lefebvre, Raymond Tsang, Rachid Amini, Vladimir Gilca, Monique Douville-Fradet, France Markowski, Philippe De Wals

**Affiliations:** 1 Institut national de santé publique du Québec, Quebec City, Quebec, Canada; 2 Public Health Research Unit, Quebec University Hospital Centre, Quebec City, Quebec, Canada; 3 Department of Social and Preventive Medicine, Faculty of Medicine, Laval University, Quebec City, Quebec, Canada; 4 Laboratoire de Santé Publique du Québec, Institut national de santé publique du Québec, Sainte-Anne-de-Bellevue, Quebec, Canada; 5 National Microbiology Laboratory, Public Health Agency of Canada, Winnipeg, Manitoba, Canada; 6 Ministry of Health and Social Services, Quebec, Canada; The Australian National University, Australia

## Abstract

**Background:**

In order to inform meningococcal disease prevention strategies, we analysed the epidemiology of invasive meningococcal disease (IMD) in the province of Quebec, Canada, 10 years before and 10 years after the introduction of serogroup C conjugate vaccination.

**Methodology:**

IMD cases reported to the provincial notifiable disease registry in 1991–2011 and isolates submitted for laboratory surveillance in 1997–2011 were analysed. Serogrouping, PCR testing and assignment of isolates to sequence types (ST) by using multilocus sequence typing (MLST) were performed.

**Results:**

Yearly overall IMD incidence rates ranged from 2.2–2.3/100,000 in 1991–1992 to 0.49/100,000 in 1999–2000, increasing to 1.04/100,000 in 2011. Among the 945 IMD cases identified by laboratory surveillance in 1997–2011, 68%, 20%, 8%, and 3% were due to serogroups B, C, Y, and W135, respectively. Serogroup C IMD almost disappeared following the implementation of universal childhood immunization with monovalent C conjugate vaccines in 2002. Serogroup B has been responsible for 88% of all IMD cases and 61% of all IMD deaths over the last 3 years. The number and proportion of ST-269 clonal complex has been steadily increasing among the identified clonal complexes of serogroup B IMD since its first identification in 2003, representing 65% of serogroup B IMD in 2011. This clonal complex was first introduced in adolescent and young adults, then spread to other age groups.

**Conclusion:**

Important changes in the epidemiology of IMD have been observed in Quebec during the last two decades. Serogroup C has been virtually eliminated. In recent years, most cases have been caused by the serogroup B ST-269 clonal complex. Although overall burden of IMD is low, the use of a vaccine with potential broad-spectrum coverage could further reduce the burden of disease. Acceptability, feasibility and cost-effectiveness studies coupled with ongoing clinical and molecular surveillance are necessary in guiding public policy decisions.

## Introduction

*Neisseria meningitidis* causes both epidemic and endemic invasive meningococcal disease (IMD) worldwide. The overall incidence of meningococcal disease in North America in the last decades has been of 1–2 per 100,000 population, with the highest age-specific incidence rates reported in children under 5 [1]. In Canada, the majority of IMD has been due to serogroup B and serogroup C over the last 20 years. Serogroup B has been associated with sporadic cases [2], while outbreaks have been associated with virulent serogroup C clones [3]. In the province of Quebec, outbreaks caused by the serogroup C occurred in the early 1990s [4], [5] and in 2001 [6]. The first outbreak triggered a mass immunization campaign using polysaccharide vaccines and targeting the population 6 months to 20 years of age in 1992–1993, whereas a monovalent serogroup C conjugate vaccine was used in 2001, targeting the population 2 months to 20 years of age [5], [7], [8]. Since 2002, one dose of serogroup C conjugate vaccine is offered at age 12 months. Vaccination coverage by 24 months of age is estimated at 96% [9]. Currently, the introduction of an adolescent booster dose as recommended by the National Advisory Committee on Immunization (NACI) is under discussion in Quebec [10].

The two serogroup C outbreaks in Quebec were caused by a unique genetic variant of the *N. meningitidis* sequence type (ST)-11 clonal complex (electrophoretic type (ET)-15, a genetic variant of the ET-37) [4], [6]. This clone was first detected and identified in Canada in 1986 and was described as more virulent than other members of the same clonal complex [11], [12], [13]. While *N. meningitidis* strains that cause outbreaks are highly genetically related, meningococci causing sporadic disease present considerable genetic diversity [2], [14]. Almost all serogroup C meningococci that caused an increase in IMD incidence in different provinces of Canada in 2001 also belonged to the ST-11 clonal complex (common or original ET-15/ET-37) [15]. Among serogroup B isolates circulating in Canada in the 1990s, no particular clone or strain was predominating [2]. However, a particular strain, the ST-269, was identified as the strain responsible for the cluster of serogroup B IMD which occurred in 2004–2005 in two adjacent regions (Chaudiere-Appalaches and Quebec City) of the province of Quebec [16]. Retrospective analysis from 2000 onwards showed that this strain first emerged in Quebec in 2003 [16]. In subsequent years, the percent of serogroup B IMD isolates that belonged to the ST-269 clonal complex (ST-269cc) increased from 35% in 2003 to 76% in 2010 [17].

At least two subcapsular protein-based serogroup B meningococcal vaccines that might provide cross-protection against a large variety of meningococci are in late phase development [18]. In order to inform future meningococcal disease prevention strategies, we analysed epidemiology of IMD cases in Quebec from 1997 through 2011.

## Methods

### Ethics Statement

This study was carried out under a mandate of surveillance and evaluation provided by the Quebec Ministry of Health and Social Services. Depersonalized data from notifiable disease registry and laboratory surveillance were used, and approval of an ethics committee was not required.

### Notifiable Disease Registry

In the province of Quebec (population 7.9 million), any IMD case suspected or diagnosed by a clinician or a laboratory has to be reported to the regional public health department. An investigation is conducted by the regional health authority, including the collection of information on disease outcome and results of diagnostic tests. Confirmed cases are those with a clinical picture compatible with IMD and *N. meningitidis* identified by culture or by PCR from a normally sterile site. Clinical cases are those with a clinical picture compatible with IMD and a *purpura fulminans* present or meningococcal-specific antigens identified in the cerebrospinal fluid (CSF). Confirmed and clinical cases along with demographic and clinical data are reported to the provincial registry of notifiable diseases. Reported cases that occurred between January 1, 1991 and December 31, 1996 have been included for historical overview. Data from January 1, 1997 through December 31, 2011 were used for the validation of laboratory surveillance data and for lethality analysis.

### Laboratory Surveillance

Since 1997, hospital laboratories have systematically been sending *N. meningitidis* isolates from IMD cases to the Quebec public health laboratory (Laboratoire de Santé Publique du Québec (LSPQ)) as part of provincial laboratory surveillance for further analysis and characterization. Serogrouping was done by bacterial agglutination using rabbit antisera. Additional tests were performed by the National Microbiology Laboratory (NML) of the Public Health Agency of Canada in Winnipeg, including multilocus sequence typing (MLST) [16]. Since 2001, culture negative IMD cases have been identified and grouped by polymerase chain reaction (PCR) by the NML using protocols previously published [19], [20], [21]. Assignment of serogroup B isolates from culture-confirmed cases from 2003 through 2011 to ST and clonal complexes was done at NML by using MLST according to the *Neisseria* MLST website (http://pubmlst.org/neisseria). Cases occurring between 1 January 1997 and 31 December 2011 have been included in the analysis. Since only <1% of IMD were of unknown serogroup and no temporal trend was observed, no adjustment was done for this.

### Statistical Analysis

IMD incidence rates were calculated by using age-, year-, and region-specific population denominators from census data provided by the Quebec Institute of Statistics [22]. Incidence rates confidence intervals (CI) were calculated by using normal approximation. P-values for trends were calculated by using Cochran-Armitage test. Historically, IMD incidence in 3 of the 18 socio-sanitary regions of Quebec has been consistently higher than in the other regions. Therefore, these 3 adjacent regions (Chaudiere-Appalaches, Quebec City, and Saguenay-Lac-Saint-Jean) are presented separately. Since assignment of serogroup B isolates to ST was performed only in culture-confirmed cases, weighted proportion and incidence were calculated for the clonal complexes to account for missing data among the PCR-confirmed cases. In order to capture the 2001–2002 serogroup C epidemic and to reflect recent trends, the 15 study years were divided in 4 periods: pre-epidemic (1997–2000); serogroup C epidemic (2001–2002); post-epidemic (2003–2008), and recent (2009–2011). Estimation of factors associated with increased IMD incidence was done by using multivariate negative binomial regression. Risk of death in IMD cases reported to the registry of notifiable diseases was assessed by using multivariate logistic regression. Strata with the lowest incidence and the lowest case-fatality ratio (CFR) were used as reference. Statistical analysis was performed with SAS (version 9.2; SAS Institute, Cary, NC, USA).

## Results

From January 1997 through December 2011, a total of 1028 IMD cases were reported to the notifiable disease registry, and 945 IMD cases to the laboratory surveillance (Table 1). Of the 945 laboratory surveillance IMD, 820 (87%) were only culture-confirmed, 125 (13%) were only PCR-confirmed, and 10 (1%) were confirmed by both culture and PCR. Overall discrepancy between notifiable disease registry and laboratory surveillance has been relatively stable over the study period (on average 8% more cases notified in the notifiable disease registry). The discrepancy was even smaller for serogroup B (1%) and serogroup C (4%) and the difference is mostly explained by clinically diagnosed cases. Among the laboratory surveillance specimens, less than 1% of IMD cases were unassigned to a known serogroup (a total of 8 during the 15 years). Proportion of PCR-diagnosed cases doubled in recent years compared to 2001 (from 12% in 2001 to 24% in 2011) (Table 1). From 39 to 101 (average 69) cases were reported annually to the notifiable disease registry and 30 to 95 (average 63) cases to the laboratory surveillance. Among IMD cases identified by the laboratory surveillance, 68%, 20%, 8%, and 3% were due to serogroups B, C, Y, and W135, respectively (Table 1). Serogroup B predominated throughout the entire study period, with the exception of 2001, when serogroup C was the most prevalent (Table 1).

**Table 1:** Yearly number of IMD cases from the notifiable disease registry and laboratory surveillance.

	1997	1998	1999	2000	2001	2002	2003	2004	2005	2006	2007	2008	2009	2010	2011	overall
**Notifiable disease registry**	**70**	**39**	**36**	**36**	**101**	**70**	**58**	**72**	**75**	**88**	**91**	**70**	**69**	**70**	**83**	1028
**Laboratory surveillance**	**63**	**30**	**35**	**30**	**95**	**68**	**56**	**67**	**74**	**77**	**81**	**62**	**65**	**64**	**78**	945
PCR-diagnosed					11	7	8	10	10	13	9	10	12	16	19	125 (13%)
serogroup B	39	22	22	22	28	31	34	45	52	57	62	46	58	55	69	642 (68%)
serogroup C	12	3	6	4	58	26	12	17	13	17	8	6	1	2	1	186 (20%)
serogroup Y	11	4	4	4	7	9	7	3	2	2	5	5	1	5	3	72 (8%)
serogroup W135		1	1		1		1	2	6	1	5	5	3	1	3	30 (3%)
serogroup X						1							1		1	3 (0.3%)
serogroup 29E			1						1		1					3 (0.3%)
serogroup Z	1															1 (0.3%)
not assigned*	0	0	1	0	1	1	2	0	0	0	0	0	1	1	1	8 (1%)

* Among the 8 IMD cases not assigned to a serogroup, one had an insufficient sample volume; 1 was PCR-diagnosed (*N. meningitidis*-specific *ctrA* and *crgA* genes positive; negative for specific A, B, C, X, Y, W135 and 29E serogroup genes); 4 were not assigned by PCR for serogroups B, C, Y and W135; and 2 were not assigned by agglutination with rabbit serogrouping antisera.

**Figure 1:**
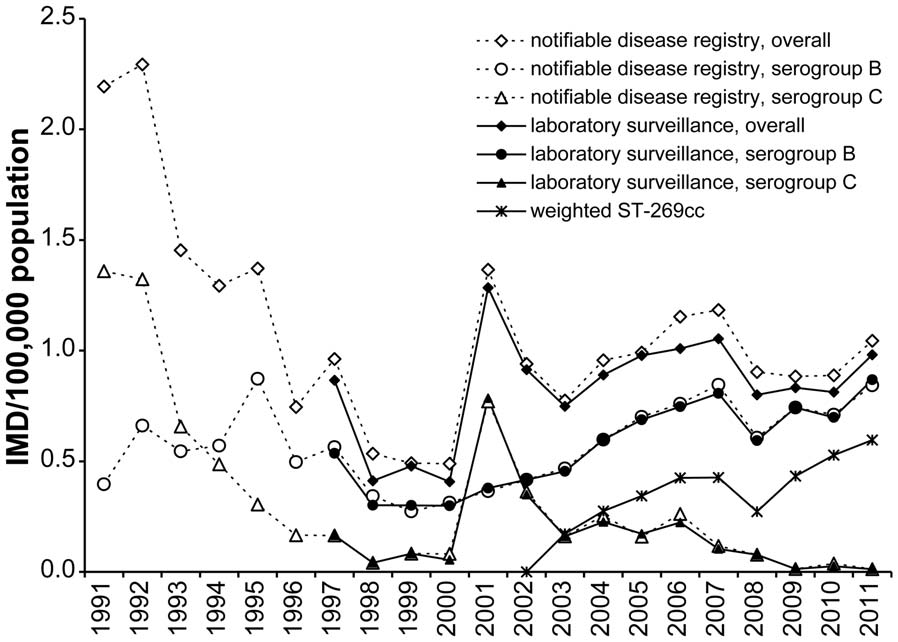
IMD incidence in Quebec, 1991–2011.

During 1997–2011, the overall annual incidence was of 0.91 per 100,000 population according to the notifiable disease registry. Historically, yearly overall incidence rates ranged from a high of 2.2–2.3/100,000 in 1991–1992 to a low of 0.49/100,000 in 1999–2000 (Figure 1). Historical highs for serogroup C-specific incidence were observed during the two epidemics (1.36/100,000 in 1991 and 0.77/100,000 in 2001); serogroup C IMD almost disappeared with only 4 cases during the last 3 years, diagnosed exclusively in adult patients (>30 years). Historical high for serogroup B-specific incidence was observed in 1995 (0.87/100,000), followed by a historical low (0.27/100,000) in 1999, then by a progressive increase, almost reaching the historical high (0.84/100,000) in 2011.

**Figure 2:**
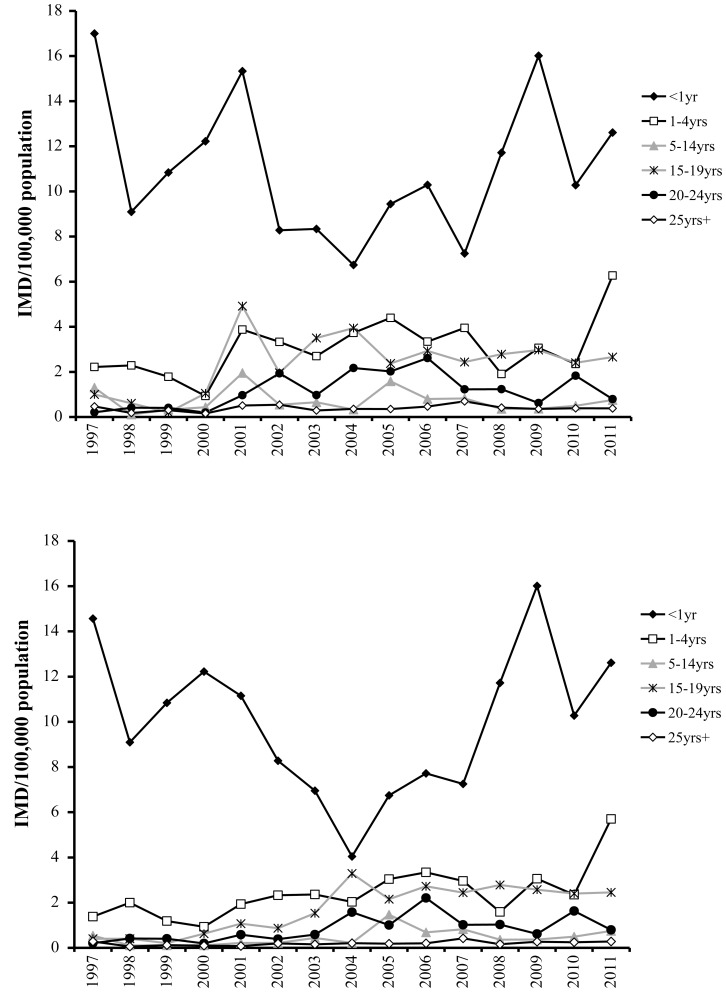
IMD incidence by age group in Quebec according to laboratory surveillance, 1997–2011. A. Overall IMD incidence. B. Serogroup B incidence.

According to laboratory surveillance, overall, almost half (47%) of IMD cases were in young children and adolescents (29% in children <5 years of age (15% in <1year; 14% in 1–4 years) and 18% in 15–19-year-olds). The highest incidence was consistently observed in <1 year-olds, followed by 4-fold lesser incidences in 1–4-year-olds and in 15–19-year-olds (Figure 2A). Increases in serogroup B incidence have been observed in all age groups, with the exception of youngest (<1 year-olds) (Figure 2B). Earliest increases occurred in 15–19 and 20–24-year-olds (2003–2004), followed by 1–14-year-olds (2005–2006), and >25-year-olds (2007). Over the last 3 years, serogroup B was responsible for all (35/35) laboratory-confirmed IMD in <1 year-olds, 94% (102/108) in 1–24-year-olds, 76% (34/45) in 25–64-year-olds and 58% (11/19) in those ≥65 year-old.

**Figure 3:**
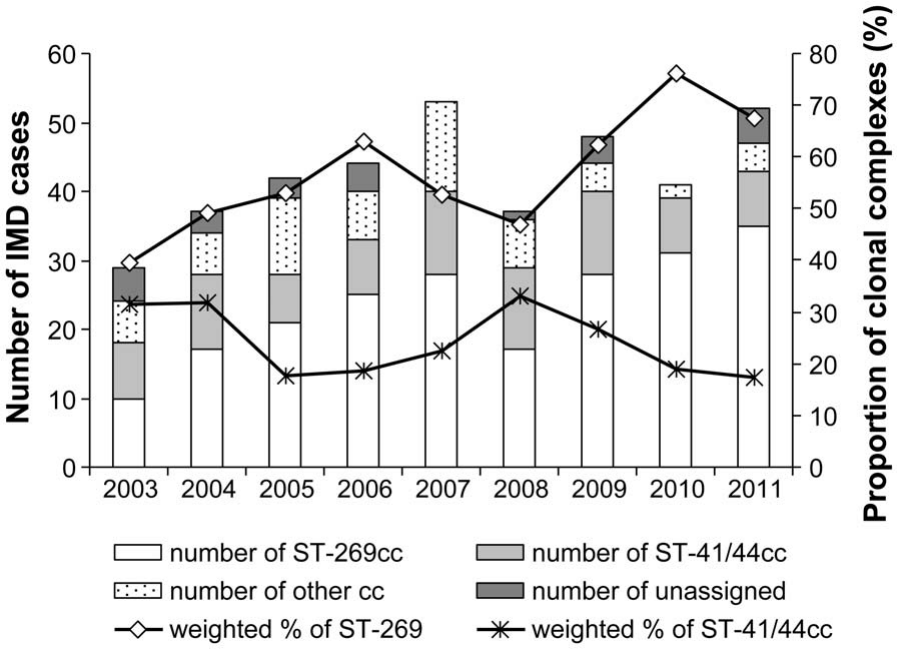
Number and proportion of different clonal complexes among culture-positive serogroup B isolates, 2003–2011.

The number and proportion of *N. meningitidis* ST-269cc among the identified clonal complexes of serogroup B IMD has been steadily increasing since its first identification (34% in 2003 to 65% in 2011, p value for trend  = 0.0004) (Figure 3). This trend was associated with an increase in estimated weighted ST-269cc incidence, which reached 0.6/100,000 in 2011 (Figure 1). The ST-269cc was first identified in Chaudiere-Appalache and Quebec City regions, and shortly thereafter in Saguenay-Lac-Saint-Jean region, and remained clustered to these three regions that also present the highest incidences in the province of Quebec. During 2003–2011, the proportion of ST-269cc among serogroup B IMD isolates was 71% in Chaudiere-Appalaches, 79% in Quebec City, and 87% in Saguenay-Lac-Saint-Jean, compared to the overall provincial 55%. The proportion of the second most prevalent clonal complex, ST-41/44, has been relatively stable in Quebec during the 2003–2011 period (variations between 16% and 32%, p for trend>0.05) (Figure 3).

In the first five years of ST-269cc identification (2003–2008), 15–19 year-olds were the most affected with 30% (n = 36, 1.26/100,000) of confirmed ST-269cc IMD cases, compared to 15% (n = 19, 0.84/100,000) in <5-year-olds. In the last three years, the situation changed: young children are mostly affected by ST-269cc, with 33% (2.08/100,000) of cases in <5-year-olds (4.58/100,000 in <1 year-olds), followed by 15–19-year-olds, with 25% (1.47/100,000) of cases.

In multivariate analysis, factors associated with increased IMD incidence were younger (less than 25 years) and older (≥65 years) age groups compared to age 25–64, study periods 2001–2002, 2003–2008 and 2009–2011 compared to 1997–2000 period, and geographical localisation (each of the three north-central regions compared to the rest of the province) (Table 2).

**Table 2:** Factors associated with increased IMD incidence in multivariate negative binomial regression, laboratory surveillance data,1997–2011.

	no IMD	IMD/100,000 (95% CI)	Adjusted RR*	95% CI
Study periods				
1997–2000	158	0.54 (0.46–0.620	1	
200–2002	163	1.10 (0.93–1.27)	2.24	1.63–3.08
2003–2008	417	0.91 (0.83–1.00)	1.94	1.45–2.59
2009–2011	207	0.88 (0.76–0.99)	1.73	1.24–2.41
Age groups				
<1yr	131	7.55 (6.26–8.84)	31.85	21.83–46.47
1–4 years	147	2.08 (1.75–2.42)	9.40	6.45–13.70
5–14 years	96	0.51 (0.41–0.61)	2.38	1.61–3.53
15–19 years	171	1.69 (1.44–1.94)	8.45	5.88–12.15
20–24 years	88	0.85 (0.67–1.03)	3.38	2.26–5.05
25–64 years	212	0.24 (0.21–0.27)	1	
65+ years	100	0.49 (0.39–0.58)	1.77	1.19–2.62
Geographical localisation				
Chaudiere-Appalaches	71	0.86 (0.66–1.06)	1.51	1.09–2.11
Quebec	140	1.01 (0.84–1.18)	1.88	1.42–2.49
Saguenay	95	1.60 (1.27–1.92)	2.96	2.18–4.02
rest of the province	639	0.50 (0.46–0.54)	1	

* adjusted for all the variables listed in the table.

### Case Fatality Ratio (CFR)

There were 84 deaths reported during the study period, with an overall CFR of 8%: 15% for serogroup C; 6% for serogroup B; 13% for unassigned serogroups (Table 3). There were 12 deaths due to ST-269cc, giving a CFR of 6%. Overall CFR was higher in ≥65-year-olds (18.9%), in 25–64 year-olds (10.1%), and in children <1-year-old (10.0%). No significant CFR differences in time were observed. The majority of deaths during the last three years were due to serogroup B (61%). In the multivariate analysis, risk factors for increased CFR were extremes of age (younger (<1 year) and older (≥65 years)), two of the three north-central regions (Chaudiere-Appalaches et Saguenay-Lac-Saint-Jean), serogroup C, and unassigned serogroups (Table 3).

**Table 3:** Factors associated with death in IMD cases in multivariate logistic regression in IMD cases reported to the registry of notifiable diseases, 1997–2011.

	Deaths/IMD cases (CFR, %)	Adjusted OR*	95% CI
Age groups**			
<1year	14/140 (10.0)	3.15	1.11–8.92
1–4 years	9/162(5.6)	1.2	0.40–3.55
5–14 years	6/115(5.2)	1	
15–19 years	8/180(4.4)	1.00	0.33–3.06
20–24 years	4/96(4.2)	0.94	0.25–3.53
25–64 years	23/228(10.1)	2.2	0.85–5.71
65+ years	20/106(18.9)	6.2	2.24–17.22
Study periods			
1997–2000	14/181(7.7)	1	
2001–2002	17/171(9.9)	1.01	0.44–2.31
2003–2008	30/454(6.6)	0.89	0.45–1.79
2009–2011	23/222(10.4)	1.78	0.84–3.74
Serogroups			
B	37/651(5.7)	0.90	0.39–2.04
C	29/194(15.0)	3.69	1.56–8.74
Not assigned	8/61(13.1)	3.01	1.02–8.89
Other serogroups	10/122(8.2)	1	
Geographical localisation***			
Chaudiere-Appalache	9/76(11.8)	2.37	1.07–5.26
Quebec	11/154(7.1)	0.91	0.44–1.86
Saguenay	11/102(10.8)	2.18	1.04–4.57
rest of the province	53/694(7.6)	1	
overall	84/1028(8.2)		

CFR, case-fatality ratio.

OR, odds ratio.

* Adjusted for all the variables listed in the table;

** There was one case with missing age information;

*** There were two cases with missing information on geographical location.

## Discussion

With the exception of a few geographically confined outbreaks, the overall incidence of IMD has decreased between the late 1990s and 2006–2007 in the USA and most European countries [23], [24]. This is mainly due to the decrease of serogroup C IMD following the introduction of serogroup C vaccination. Overall IMD incidence in Quebec presented an increasing trend during the same period and a certain stabilisation during 2007–2011. Although this new level (1/100,000) is lower than that observed in the pre-vaccination period, it is 2 times higher than the incidence in other Canadian provinces (0.47/100,000 in 2008) and in the USA (0.28/100,000 in 2009) and is comparable to the incidence in Europe (overall 0.92/100,000 in 2009) [25], [26], [27]. However, the IMD incidence in Quebec remains lower than in Ireland (3.4/100,000 in 2009) and the UK (2.0/100,000 in 2009) [27].

Following the 2001 mass immunization campaign and the introduction of a routine one-dose monovalent C conjugate vaccine program for 12-month-old children in 2002, serogroup C IMD has been virtually eliminated with only a few cases in older patients. Meanwhile, serogroup B incidence has been steadily increasing since the historical low in 1998–2000. In the last three years, serogroup B has been responsible for 88% of all IMD cases reported to laboratory surveillance and 61% of all IMD deaths. Only a few cases due to serogroups C, Y and W-135 have been reported and serogroup A was absent. This means that the great majority of IMD cases and related deaths in Quebec are not vaccine preventable using currently available vaccines.

Among serogroup B IMD, 2/3 of cases are now caused by ST-269cc. The proportion of this clonal complex among serogroup B IMD isolates and its incidence, which is paralleling the serogroup B IMD incidence, has been continually increasing since 2003 and is estimated to be accounting now for more than half of overall IMD incidence in Quebec. In other Canadian provinces, ST-269cc is less common. For example, in the neighbouring province of Ontario, only 20 (10.4%) of the 193 invasive serogroup B isolates collected from 2001 to 2010 belonged to ST-269cc. In provinces other than Quebec and Ontario, of the 46 serogroup B isolates recovered from IMD cases in 2006 to 2009, only 7 (15.2%) belonged to ST-269cc (Public Health Ontario and NML, unpublished data).

ST-269cc was first reported in 1975 in England, and since then it has been identified in the USA, France, Belgium and Japan [28], [29], [30], [31]. Although localized ST-269cc outbreaks [29], [30] and some increases in the number and proportion of ST-269cc were observed over time [32], [33], [34], reported increases have not been as dramatic as in Quebec in terms of rapidity and magnitude, neither has ST-269cc become a predominant clone. Overall prevalence of this strain in other parts of the world remains low, with the highest proportion of ST-269cc (36%) among all IMD having been reported in England and Wales between 2006 and 2010 [35]. Another particularity of Quebec ST-269cc epidemiology is that its population is highly homogenous, with a great majority (92%) of the meningococci in ST-269cc belonging to a single sequence type (ST-269) [17]. This is different from other provinces of Canada and other parts of the world, where the ST-269cc population is more heterogenous [17], [36]. We can not exclude that epidemiological changes in Quebec are due to natural cyclical patterns of meningococcal disease. However, the increase in ST-269cc incidence, ST-269cc predominance among serogroup B IMD cases, its homogeneity and specific age distribution could suggest that the ST-269cc may however represent an emerging clonal complex of meningococci.

Previous reports show that a limited number of clonal complexes have been responsible for the majority of IMD reported during the 20^th^ century. As an example, serogroup B ST-11cc (ET-37) caused an epidemic in the US Army in the 1960s [37]; a new variant member of the ST-11cc (ET-15, serogroup C) caused an increase in IMD in Canada in 1986 [4], was later associated with outbreaks in the USA [38] then spread throughout Europe [37], leading a number of countries to introduce serogroup C conjugate vaccines into routine immunization programs. Variants of the ST-32cc (ET-5), usually expressing serogroup B capsules, have been responsible for epidemics in Norway and Spain in the 1970s, followed by increases in IMD incidence and outbreaks in other European countries [39]. Members of the ST41/44cc displaced the ST-32cc as the major cause of serogroup B IMD in Europe during the 1990s [40]. Although the ST41/44cc was associated with an increase in serogroup B IMD in the Netherlands starting in 1980 [41], it has not been associated with large outbreaks in other European countries. However, a particular variant of the ST41/44cc was responsible for a long-lasting epidemic of IMD in New Zealand, where it represented more than 70% of serogroup B isolates in 1995 [42].

The genetic and antigenic differences of meningococcal isolates, diversity in their pathogenicity, phenotypic expression, as well as interstrain competition, mediated by immune selection, may explain some differences in sporadic, endemic, or epidemic occurrence of meningococcal disease [43], [44], [45]. However, at this point meningococcal disease epidemiology remains highly unpredictable. Other factors, such as host (age, low level of serum bactericidal antibody, ethnicity, genetic polymorphisms, deficiencies in innate immune system components) and environmental (overcrowding, behavioural risk factors, low socioeconomic status) have been shown to be associated with risk of IMD [46], [47]. Complex interplay between the pathogen, the host and environmental factors most probably contribute to spatiotemporal variations in disease incidence and severity.

In Quebec, changes in serogroup B age distribution with incidence increase first seen in 15–24-year-olds, followed by the younger and older age groups, corresponded to ST-269cc spread. We hypothesize that this clone was first introduced in adolescent and young adults and then spread to other age groups, analogous to the pattern observed in epidemic settings. First cases were detected in three adjacent regions and remained clustered thereafter. IMD incidences in these three regions, as well as CFR in two of them, were consistently higher than in the rest of the province. Causes of this clustering remain unknown. Deprivation indexes in these regions are comparable to other parts of the province and it is unlikely that ethnicity or overcrowding played a major role. This is different from the New Zealand epidemic, which affected mainly Maori and Pacific populations and was also associated with household crowding [42]. We can not, however, exclude that other variables, such as genetic factors shown to determine human susceptibility to meningococcal infection and its outcome [46], have played a role. In the three most affected regions of Quebec, the population is genetically more homogenous compared to the rest of the province. At least one of the three regions (Saguenay-Lac-St-Jean) is known for an increased proportion of genetically inherited diseases [48].

Although members of ST-269cc have been reported to be more likely associated with disease than with carriage [49], [50], to our knowledge, no data exist as to higher CFR associated with this particular clonal complex. Our results show an increase in the incidence associated with ST-269cc, but not a higher CFR associated with serogroup B (or ST-269cc). This is consistent with data from England and Wales, where serogroup B (with around a third belonging to ST-269cc) presented a CFR close to that observed in our study (5.2%) [35]. It is possible that higher transmissibility, not pathogenicity, explains predominance of a limited number of STs in meningococcal populations [44], [45].

Limitations of our study include the absence of an unique identifier between the two sources of data and missing information on clinical data and sequelae among cases reported to the notifiable disease registry. This precludes us from presenting clinical data and outcomes in this analysis. A study aiming to validate notifiable disease registry data with clinical files data in Quebec is ongoing. However, we think data on deaths are valid since this outcome is generally well reported, and there is excellent correspondence between deaths reported to the notifiable disease registry and to the provincial death registry. The increase in overall and serogroup B-specific IMD incidence based on laboratory surveillance data during the last years may be partially explained by a potential ascertainment bias due to an increasing number of PCR-confirmed cases. However, we believe this has not impacted on the overall incidence based on notifiable disease registry data since it is unlikely that reporting of clinical cases by physicians has changed.

In conclusion, important changes in the epidemiology of IMD have been observed in the province of Quebec during the last two decades. Serogroup C meningococci virtually disappeared since the implementation of a universal childhood immunization program. During the last years, most cases have been due to the serogroup B ST-269cc. Although overall IMD incidence somewhat increased in recent years, it remains relatively low, with important variations across regions. Different options for the use of future protein-based serogroup B meningococcal vaccines may be considered. They might be included in the routine immunization program, or as a targeted intervention in regions/populations at higher risk. A routine immunization program is not likely to be economically attractive, given the relatively low current level of incidence. However, the high level of public concern about meningococcal disease and its sequelaes may outweigh economical considerations. Recent data show that probability of long-term sequelaes after serogroup B IMD may be more important than previously estimated [51]. Incorporation of these parameters into cost-effectiveness modelling may change current estimates. Furthermore, given historical dynamics of IMD epidemiology, it is unpredictable whether low disease incidence will persist and what potential regional or global impact emerging strains may have. It is not fully understood whether new multicomponent vaccines against serogroup B meningococci will protect against particular strains circulating in different regions, and how long the protection will last. In addition, it is not known what impact the new vaccine will have on the dynamic of nasopharyngeal carriage and consequently on herd protection. Acceptability, feasibility and cost-effectiveness studies coupled with ongoing strengthened clinical and molecular surveillance of IMD should be assessed when prioritizing, recommending, and evaluating meningococcal disease prevention and control strategies.
